# Past as Prologue: Predicting Potential Psychosocial–Ethical Burdens of Positive Newborn Screens as Conditions Propagate

**DOI:** 10.3390/ijns10010012

**Published:** 2024-02-06

**Authors:** Lynn W. Bush, Harvey L. Levy

**Affiliations:** 1 Department of Medicine, Division of Genetics and Genomics, Boston Children’s Hospital, Boston, MA 02115, USA; harvey.levy@childrens.harvard.edu; 2 Center for Bioethics, Harvard Medical School, Boston, MA 02115, USA; 3 Department of Pediatrics, Harvard Medical School, Boston, MA 02115, USA

**Keywords:** newborn screening, psychological, psychosocial, positive, ethical, ELSI, attachment, anxiety, stress, depression

## Abstract

We look to the past as prologue for guidance in predicting and circumventing potential psychosocial–ethical challenges, including those that may influence the attachment process for some parents. We consider the evolution of bioethics and developmental psychology as they intersect with newborn screening while exploring potential implications of positive findings, be they false positives, true positives, or secondary as well as incidental findings. We reflect on navigating the complex landscape that may be significantly impacted by variable phenotypes, the age of onset, and uncertain prognoses, mindful of the diagnostic odyssey *continuum*. We explore select facets of ethical and psychological challenges encountered with positive newborn screening findings by highlighting enduring debates to improve the policy process in public health and medicine. We believe substantive empirical research is needed, including long-term follow-up, routine prenatal assessment of tolerance for uncertainties, and especially innovative methodologies to better evaluate potential psychological distress that may be present in some at-risk individuals during the perinatal period preceding and following reports of positive findings. Mitigation strategies building on lessons learned from NBS and clinical follow-up should be implemented and studied. We conclude by pondering why we remain far afield from providing these services. Research directed towards understanding the implications of positive NBS findings will further reduce the burdens on families and care providers alike and should lead to improved communication.

## 1. Introduction

We look to the past as prologue for guidance in predicting and circumventing potential psychosocial–ethical challenges, including those that may influence the attachment process. We consider the evolution of bioethics and developmental psychology as they intersect with newborn screening while exploring potential implications of positive findings, be they false positives, true positives, or secondary/incidental findings. We reflect on navigating the complex landscape that may be significantly impacted by variable phenotypes, the age of onset, uncertain prognoses, and the increasing prevalence of identifying and disclosing carrier status [1]. We consider this landscape while being mindful of the ‘diagnostic odyssey *continuum’*, a term informally used by, and likely coined by, one of us (LB) “since the 1980s in the context of how families navigate the uncertain course of how their child is ‘labeled’—from presumptive diagnosis, a confirmatory diagnostic ‘label’, and uncertainties in prognosis and management [2]” (p. 7).

Other factors of import to be considered include balancing the best interests of the child with the interests and values of the family and society as well as the blurring of distinctions between health, illness, “dis-ability”, severity, “normalcy”, and rarity. Consideration must also be given to the fact that alongside identification of risk exists ascertainment bias, particularly since the diversity of the population is not yet adequately reflected in the variant databases. Important too is selecting criteria decision-points for expanding conditions using new technologies to reduce the diagnostic odyssey *continuum* and to inform management, with keen appreciation for new therapies today and on the near horizon [3]. 

We believe substantive empirical research is needed, including long-term follow-up, routine prenatal assessment of tolerance for uncertainties [4,5], and especially innovative methodologies to better evaluate potential psychological distress that may be present in some at-risk individuals during the perinatal period. This vulnerability is important to assess both preceding and following reports of positive findings. Mitigation strategies building on lessons learned from NBS and clinical follow-up should be implemented and similarly studied [6,7,8,9]. 

We explore select facets of ethical and psychological challenges encountered with positive newborn screening findings, largely within the United States (US). We purposefully limited the scope of the issues covered in this opinion piece, choosing to highlight some enduring debates that may be unfamiliar to some or long-forgotten. This historical reflection aims to enhance our prediction of potential burdens as NBS conditions propagate and to raise predictions of efforts that will be necessary to reduce such potential burdens and to maximize potential opportunities.

Conflicts have been prevalent to varying degrees across the globe since NBS’s inception, often in response to resource limitations and priorities. In contrast to much of the world, where NBS is optional or non-existent, conflicts arise in the US in part because of the country’s mandatory NBS policy. Justification for statewide compulsory testing without parental informed consent tugs at some of our most sacred ethical principles—pitting non-maleficence against respect for a parents’ autonomy (more precisely, parental “authority”) on behalf of their child. Historically, as the early years of PKU screening overlapped with the emerging field of bioethics in the US, tensions mounted in response to mandated population screening juxtaposed with their increasing value of autonomous rights. 

In parallel, concerns accelerated with expanded screening practices enabled by technological advances—notably tandem mass spectroscopy (MS/MS) and genomic testing—further provoking debate among stakeholders. It is not insignificant that, even today, parents are oft-times not cognizant that screening is being done on their infant until after the fact. Moreover, parents tend to be poorly informed ahead of time that subsequent testing may be required to evaluate a screening result and that it is important to pursue additional diagnostic testing if recommended. 

This lack of adequately communicating information and counseling extends to explaining positive results. The deficits surrounding the disclosure of findings, whether true-positives or false-positives (which may signify carrier status), must be better understood to most effectively address the mounting challenges that individuals and the public health system will face in this genomic NBS era. Particularly, we need strategies that are sensitive to the fact that the return of NBS results can potentially elevate emotional distress for *some* individuals even when the final findings are benign—which is especially challenging when involving the public health domain [10]. 

## 2. The “Vulnerable Child Syndrome” Is Postulated

Around the same time that NBS for PKU began in Massachusetts, Hartmann [11] stressed the prognostic importance of infant vulnerability and risk by considering an interactional matrix of constitutional, maturational, and ecological factors. He proposed a framework regarding the relationship between vulnerability and the cumulative trauma of environmental and developmental stressors along the nature vs. nurture continuum. 

Of great significance to this present review of NBS and the attachment process—and we believe often misinterpreted and sensationalized—is Green and Solnit’s [12] 1964 analysis of what they termed the “vulnerable child syndrome.” Their reference point centered on *very severe pediatric illnesses* and parents who anticipated that their children would experience an *appreciably premature death*, not unlike the scenario of fear and uncertainty with positive or equivocal NBS results for *some* conditions. It is essential, however, to appreciate the context that grounds Green and Solnit’s findings. They noted the psychological impact on the attachment process and developing child *if* a parent had *persistent mourning* that continues *long after the real threat of loss resolved*. Importantly, they furthermore noted the benefits that were observed when they recommended therapy for the parent as well as the child. 

Green and Solnit clearly emphasize “the relief which both child and parents experience when the problem becomes clarified through verbalizations” (p. 58) in the very first paragraph of their now-famous article. However, to date, NBS research often mistakenly highlights Green and Solnit as if they posited a fait accompli of long-term pathological reactions by *most* parents to the threatened loss of a child, with the consequence always equated to “disturbance in psycho-social development” in the child. Furthermore, by viewing false positives as a universal expectation which is both deleterious and unresolvable, little weight is given to Green and Solnit’s equally important articulation of the relative ease of therapeutic intervention through early follow-ups and the preventative measures that may be taken by medical professionals after the critical period is over. They endorse interventions such as minimizing retrospective comments focusing on the tenuousness of the predicament now past, or refraining from underestimating the need to clarify that the child is now “physically sound” (p65). From an ethical standpoint, not enough attention is given by NBS researchers to Green and Solnit’s early cautionary advice that emphasis must be quickly relayed, and repeated, to all parents that their “child will not be more vulnerable than other children to illness”, particularly when “the doctor suspects that the parents may not be able to carry this off on their own, e.g., when the physician knows of predisposing factors in the family history” (p. 66). 

Having had the benefit of speaking with Solnit on many occasions in subsequent decades (including pediatric thanatology and vulnerable child conferences), my (LB) interpretation of his work suggests that many of the parental expectations, and distress, regarding possible life-threatening conditions revealed through NBS could similarly be alleviated to some degree with better communication and adequate counseling. Understanding seems particularly relevant in the context of false positive findings amongst parents with a pre-existing tendency towards heightened anxiety, to include a lower tolerance to uncertainty. In addition, greater access to treatment along with parental support and education (including concepts such as disease susceptibility and risk factors) can likewise diminish the potential impact from positive NBS for most families. 

Notably, Hartmann emphasized the impact of *sustained parental emotional absence* on young children, a circumstance that we consider to be far more severe than most instances of NBS. This growing interest in, and the developmental literature on, powerful parental influences, including those fostering the “vulnerable child syndrome”, gave rise to similar concerns in the context of NBS. 

## 3. The “PKU Anxiety Syndrome” Is Coined

Four years after Green and Solnit’s classic paper on “The Vulnerable Child Syndrome”, the oft-cited Rothenberg and Sills’ “PKU Anxiety Syndrome” [13] reported an adverse impact to the early establishment of bonding resulting from positive NBS results. While Rothenberg and Sills’ observations have merit in considering reactions to a different set of circumstances than Green and Solnit proposed, and in accumulating reports of parental grief following the delivery of a very ill or premature infant, they took a significant leap in concluding that the scenarios which they posited parallel false positives in NBS with an infant who appears to be healthy.

“Since July 1, 1966, we have seen a steadily increasing number of parents who are suffering from what we, at Bronx Municipal Hospital Center, have come to call “The PKU Anxiety Syndrome.” This syndrome presents as acute and chronic anxiety ranging in degree from mild, periodic bouts to acute anxiety hysteria. It is present in parents who persist in their belief that their babies are or will become mentally retarded [sic] despite repeatedly negative tests and considerable reassurance and support from physicians. Our current, conservative estimate is that we are seeing two to four such families a month. In essence, these parents are saying, “The test that the big Board of Health laboratory did showed that my baby was mentally retarded [sic]. Then a doctor in a little cubicle in the Clinic did a test and said it was all right. He’s reassuring—but I don’t know…”.

(p. 691)

Their caution, from our perspective, was not well grounded in developmental attachment theory. The majority of parents who have newborns with serious health problems still develop strong attachments in spite of the emotional toll. They do, however, offer some communicative, therapeutic suggestions that are strikingly similar to Green and Solnit’s, valuable preventative approaches that oft-times remain lacking, even today.

“Two major steps have been initiated: first, a pediatric resident tells every mother, after she has been with her baby and just before she leaves the hospital, that the PKU Screening Test has been done on her baby…; secondly, an intensive follow-up program has been started, in which pediatricians with psychiatric consultation provide ongoing opportunities for the parents whose babies have had false positive tests to ventilate their feelings and receive support and reassurance until their anxiety has been properly controlled. (The mothers, for example, could observe, with the pediatrician, the landmarks of their babies’ normal growth and development and see that evidence of retardation [sic] is *not* present.) We feel that the PKU experience might provide us with a model for recognizing the possibility of similar developments. As mass testing programs for the screening and treatment of inborn errors of metabolism expand they could invite further emotionally charged and possibly premature legislative response with subsequent iatrogenic emotional dysfunctions.”.

(pp. 691–692)

Critiques tend to be dichotomized to this day, often depending on whether the reviewer’s position is for or against expanded testing prior to conducting their research. 

As we reflect upon the “bonding” concerns in relation to NBS, first being raised by Rothenberg and Sills, it is important to keep in mind that the developmental theorists were considering *severe* and *very lengthy* disruptions to the attachment process that significantly impacted the child rather than the context typically following a false-positive PKU screen. Our perspective, grounded in these theories, is not meant to negate that *some* parents may themselves be more constitutionally vulnerable to anxiety or depression and hence react with greater distress under variable situations, as when given a false-positive or uncertain NBS finding. In fact, these individuals most likely need additional support and counseling, a point typically endorsed by those in the field of infant psychiatry/psychology, including Solnit. Rather, our clinical experience more closely aligns with the research that suggests the resolution of anxiety for *most* parents shortly after confirmatory negative diagnostic testing or following personal contact with a knowledgeable physician or genetic counselor. This is consistent (although it is frequently not recognized by NBS researchers) with Green and Solnit’s position paper on the vulnerable child highlighting these preventative and therapeutic management approaches. 

## 4. NBS Expands, as Does Infancy Research 

NBS began to slowly expand beyond PKU in the US, first to include congenital hypothyroidism (CH), an endocrine dysfunction resulting in significant cognitive and growth impairment unless hormone treatment is initiated in early infancy [14]. Then followed additional conditions that could be identified from a newborn blood specimen but which required a separate test for each condition. 

Around this time, two controversial bioethical issues emerged. The now-classic Baby Doe clinical ethics case involved an infant with Trisomy 21 who had esophageal atresia for which surgery on a cognitively impaired newborn was denied and then hotly debated across the media. There was also an enduring public health debate about parental autonomy/authority vs. the policy of compulsory NBS, a US policy that remains in contrast to much of the world. This debate ensued between George Annas [15] and Ruth Faden [16,17] in 1982 and revealed many psychosocial–ethical concerns that remain relevant today. It is important to note that Faden strongly supported well-informed notification and supportive services for compulsory NBS with further consideration regarding beneficence, best interest, and the harm principle. Had Faden’s position been heeded—that parents should be informed with the availability of better communication and provision for adequate follow-up “medical resources” [17] (p. 1398)—we believe much of the excessive anxiety still reported from the disclosure of all positive findings would be diminished, particularly with adequate counseling after the return of false-positives results. And notably, Annas’s prescient concern that emerging biotechnologies will result in additional screening for numerous, relatively rare, inborn errors of metabolism that may demonstrate less validity and reliability than PKU parallels tensions seen today. Controversial psychosocial implications, particularly parental distress stemming from lysosomal storage conditions and false-positive findings, continue to be cited by some researchers today.

During this period, the CF Foundation Task Force on Neonatal Screening (1983) [18] expressed concern that NBS for cystic fibrosis (CF) can negatively alter the attachment process and hinder parent–infant interactions. Farrell [19] raised concern of a potentially serious negative impact of CF NBS on the parent–child relationship. Concurrently, developmental theorists began characterizing “attachment disorders” with criteria matching very severe depressive and anxiety states, notably more severe than the milder range assessed by NBS researchers using the Parental Stress Index (PSI, a questionnaire measure) [20]. Although the level of psychological dysfunction was not aligned, nevertheless, one would hope that NBS policy be designed to proactively mandate educational and counseling interventions and identify parents who are particularly at risk—in the interest of the parent and child, much like Green and Solnit, Rothenberg and Sills, and Faden recommended.

Describing the importance of examining the psychological manifestations of high-risk children, Drotar, in 1986 [21], suggests studying the long-term impact of interrelationships among symptom formation, compromised family systems, inherent vulnerabilities, and early parent–child interactions. In fact, it is just such longitudinal studies that are typically lacking in NBS research that conclude significant interference with the attachment process, which is suggestive of long-standing chronicity—although they do not measure the outcome over sufficient time and tend to use relatively small sample sizes as well as failing to assess prenatal psychological factors. 

## 5. Tandem Mass Spectrometry

The application of the multiplex serial platform of tandem mass spectrometry (MS-MS) [22] in the late 1990s introduced a marked expansion of NBS by testing for many metabolic conditions while requiring only two tests and only a couple of discs to be removed from the Guthrie blood card. Thus, MS-MS dramatically changed the paradigm of NBS from one test/one disorder to one or two tests and many disorders. However, along with potential benefits from this expansion in conditions came mounting concerns about possible adverse effects on bonding from NBS. 

Earlier in the 1990s, Clayton [23] in particular voiced increasing discomfort surrounding parental anxiety and the risk to attachment formation with the new expanded NBS. Especially prevalent were studies involving the reporting of many positive screens for Cystic Fibrosis (CF) [24], far more frequently than the known frequency of CF (an autosomal recessive condition that produces a greatly thickened mucus, causing severe lung and gastrointestinal disease). Amongst their findings, Tluczek, Mischler, Bowers et al. [25] reported that parents expressed a preference for being informed in-person by a physician that retesting was necessary, which is generally believed to reduce distress and misinformation. Notably, however, to date, the message often reported by other researchers citing this paper is that NBS creates havoc with parents’ psychological functioning and profoundly impacts bonding. Consequently, these commentators tend to believe that expanded screening should cease, rather than noting that stress can be avoided or diminished if communication of the NBS process and its findings are relayed during appointments with knowledgeable health professionals. 

## 6. Expanding Testing Expands Concerns

With expanded newborn panels becoming more common across many states, Kwon and Farrell [26] caution, in 2000, for an even greater need to improve strategies for education and communication when confirmatory tests are needed following initial positive screens. However, problematic methodology in many research designs hinders an adequate assessment of whether diminished anxiety and improved parent–infant interactions result from changes in communication. A few years later, one of us (HL) shared historical reflections for guidance, writing *Lessons from the Past--Looking to the Future. Newborn Screening* [27]. In 2003, the Secretary’s Advisory Committee on Heritable Disorders in Newborns and Children (SACHDNC) was chartered to advise the Secretary of Health and Human Services (HSS) on the expanding application of universal NBS technologies and policies, coordinated through the Maternal and Child Health Bureau, Health Resources and Services Administration (HRSA). One of the goals was considering the selection of conditions on a uniform screening panel recommended by the American College of Medical Genetics (ACMG) [28,29], lists of tests that are also used to inform both parents and health professionals and increase communication avenues to diminish psychological distress.

In 2006, Gurian, Kinnamon, Henry, and Waisbren [30] used phone interviews with the Parental Stress Index (PSI) short form on a relatively larger group (173 families) to assess parents whose newborns had false-positive metabolic disorder screening results. Increased levels of stress were reported disproportionately by mothers, 23% compared to 10% for fathers (which they note may perhaps be more related to willingness to disclose than reflective of an accurate measure of stress). They found that many families receiving false-positives did not even know the reasoning for follow-up confirmatory testing, which leads to the suspicion that improved communication and education (e.g., iteratively during pregnancy, just prior to newborn heel-stick, with the informing of positive results, and during confirmatory testing) may reduce stress for many. In comparison to the control group, 11% of the study mothers whose test results indicated levels that were not indicative of pathological stress had PSI scores in “the clinical range”. Lacking information as to the psychological status of the parents prior to receiving the NBS results, the potential exists that some of the high-stress parents began with a higher degree of vulnerability, as conceptualized earlier in the developmental literature. 

Importantly, in addition, since stress was being measured rather than depression, we are unable to conclude that an outcome of this level of stress for this discrete period of time can contribute to attachment disturbance or be correlated to severe parental depression. Examining whether disruptions to bonding are long-term, and to what degree, will necessitate significantly deeper, broader, and lengthier prospective longitudinal mixed methodologies, with larger scale qualitative and empirical measures and larger data bases. 

To assess the psychosocial implications of false-positives and communication practices in light of controversies over the expanded metabolic screening of newborns, Hewlett and Waisbren [31] examined nine published studies in the literature from 1983 to 2006. Interestingly, the “PKU Anxiety Syndrome” [13] was mentioned as the historical starting point—without questioning the analysis of Rothenberg and Sills’ findings. Importantly, Hewlett and Waisbren noted that, although there has been “recognition of the problem of false-positive results in newborn screening and subsequent long-term stress in parents for over 35 years, little if anything has changed to improve this situation”, and summarized:

“False-positive screening results have been associated with increased anxiety and stress in parents of infants who require follow-up testing, even after the infant’s good health is confirmed. The results of this review suggest that parental stress and anxiety can be reduced with improved education and communication to parents, specifically at the time of follow-up screening.”

(pp. 677–678)

Five years after Alexander and van Dyke [32] reported on their vision for the future of NBS, in 2011, Burke et al. [33] highlight epidemiologic facets of many of the tensions and deliberations “foreshadowing the complexity” (p. 149) that they posit will intensify with population genetic screening employing next-generation sequencing. They suggest that the benefits of implementing genomic screening for newborns as a public health initiative must be carefully weighed against a greater frequency of controversial findings: results that may be considered incidental (including susceptibilities to adult-onset disorders), findings with uncertain significance, findings that indicate carrier status of genetic disorders, or false-positives. With regard to false positive results:

“anxiety, depression, and parent-child dysfunction may occur among these parents. Importantly, this anxiety appears related to poor parental understanding of newborn screening. Studying the psychosocial effect of false positives is complicated by both the process and content of communication between providers and parents, including the sense of urgency with which an initial positive result is reported. Relatively few studies have been reported. Consequently, a number of unanswered questions remain about the type and scope of harms.”

(p. 152)

## 7. Bioethical Reflections: Back to The Future

Newborn screening, with all its historical debates and complexities—for example, Annas v Faden regarding consent; SACHDNC v President’s Council on Bioethics [34]—represents, from our perspective, a model paradigm to explore bioethical principles [35,36]. However, it is beyond the scope of this opinion paper to review much of the empirical research or to address the entirety of ethical concerns in the US and internationally that we believe represent the full range of moral justifications for this public health measure.

We, however, are clear as to the tenets that we deem most relevant to the current central psychosocial theme surrounding positive findings: beneficence, the best interests of the child, and the harm principle in the context of a vulnerable population and the obligations society owes these newborns. For our final reflections, we look back forty years to what we consider to remain one of the most critical ethical analyses of NBS in the US—the words of Faden and colleagues [17] (Faden, et al., 1982)—to help navigate future policy dilemmas moving from current NBS programs and genomic NBS research to the actual implementation of more comprehensive genomic NBS within a public health program.

“In this form of screening, the intervention poses minimal risk of harm to normal infants and holds promise of the remote but important benefit of preventing mental retardation [sic]… Thus, there appears to be no reasonable question or issue of judgment as to what is in the best interest of infants…To require parental consent entails an obligation to respect parental refusal, and it is the validity of such refusals that we question. If the principle consideration is the welfare of children, their welfare is best served in this case by a program of compulsory and exceptionless screening”

(p. 1397). (a US-centric position)

The underlying ethical interest in protecting the welfare of infants has its roots in the framework of the harm principle. With that aim in mind, to maximize beneficence and non-maleficence, Faden and colleagues emphasize:

“By making child welfare the overriding consideration in policy determinations about participation in child health programs, we are taking the position that in this context, any rights of parents independent of their role as advocates for their children’s welfare is subservient… contemporary ethical theory can be traced historically to John Stuart Mill’s On Liberty, where he discusses what is now popularly referred to as the harm principle…. parental refusal of PKU screening unjustifiably poses a risk of harm to the child…”.

(pp. 1397–1398)

Whilst informed consent is not offered for US-mandated NBS conditions (separate from research), the delineation of being morally obliged to *inform* even in the absence of *consent* is of great import, and particularly relevant to the enduring call for greater communication to diminish anxiety, as we address herein. The ethical significance of providing parental notification in light of no provision for parental consent is substantial, and highlighted by Faden and colleagues:

“In arguing against a right of parents to refuse neonatal screening, we make a distinction between the obligation to obtain parental consent (and respect parental refusal) and the obligation to inform parents about the procedures or interventions that are performed on their children. That parents have a right to know, even if not a right to consent, can be justified…”.

(p. 1398)

Another essential ingredient to promoting the welfare of children through US mandatory newborn screening—both from a social justice perspective and being morally obliged in our opinion given the state’s interest to mandate screening—is the provision of and ease of access to professionals who treat and counsel, immediately after an initially positive screen and for follow-ups. This remains an issue as it was four decades ago when Faden et al. declared:

“We see the state as obligated to assure that the medical resources necessary for effective treatment are made available…adequate follow-up and counseling for parents of babies with positive tests, as well as referral to pediatricians who are experienced in the management of PKU. Unless the state provides these resources, it is doubtful that the promised benefits which justified compulsory screening will be achieved.”.

(pp. 1398–1399)

In addition, we believe it is essential to clarify what exactly is meant by disruptive levels of anxiety and depression amongst parents despite learning that the infant does not have the condition previously described, psychopathology reported in many of the studies that are firmly planted in Rothenberg and Sill’s ideology. This is particularly disconcerting since no base level of psychological functioning has been undertaken for the parents prior to the infant’s birth and newborn screen for comparison. Perhaps the families most susceptible to significant distortions in the attachment process were the most vulnerable to begin with?

Interestingly, throughout those decades, many of the researchers who have been the most adamant in opposing NBS because of the emotional sequelae from false positives have also suggested that improved communication would likely ameliorate the bonding difficulties noted by parents. Given the controversy of whether parental distress actually does continue long-term or not, further research is needed to assess whether the current modes of communication—improved since the early days of expanded screens although still inadequate—have tempered the degree of parents’ reported levels of anxiety and their potential impact on the attachment process. And a salient question that needs to be reflected upon is whether, and how much, the debate focusing on the consent process deepens the communication gap? We wonder if the reluctance of health care providers to inform families ahead of time about NBS influences their decision-making about opting-out and acting on screening results, including actionable incidental findings and referrals to specialists. 

It is difficult to consider some of the early reports of *profoundly* disturbed bonding and its sequelae as being as salient today given many of the efforts, albeit not enough, that are currently made (i.e., Baby’s First Steps, March of Dimes, piloted initiatives) [37,38,39,40] to better educate the public and health professionals as to newborn screening practices. Since false-positive results are an expectable necessary risk to avoid overlooking truly affected infants, as conditions expand so too will the reporting of false positives, with an increased need for communicating and counseling some families. Furthermore, it is important to clarify that, with true positives, there will be, and at times remain, a degree of uncertainty as to the penetrance, variable outcome, and onset of each condition as well as differing phenotypic expression (some epigenetic) existing alongside genotypic similarities. However, we do not know how to best communicate NBS findings to reduce the burdens to *some* families. We need to construct more effective educational and counseling paradigms through more nuanced research that is attentive to psychosocial aspects of positive findings.

Notably, several contemporary scientific strategies are also available to reduce false positives that we believe should be implemented when feasible to minimize potential psychological burdens. Some current approaches that can be utilized to improve, albeit not fully resolve, the predictive value of NBS results reported to parents include the incorporation of more second-tier testing and probabilistic modeling that employ ratios (i.e., CLIR). Furthermore, since variants of uncertain significance in coding regions are predicted [41] to become increasingly less problematic (though not absent) over the next decade as advances are made in genomic interpretation, data sharing improves, and efforts are made for greater population diversity in the reference genome, genomics will then increasingly play a role (whether supplemental, involving large panels, or more comprehensively) in minimizing false positives and uncertain diagnoses for some conditions.

The technological developments in whole genome sequencing or whole exome sequencing present opportunities and many challenges, including those that are psychological as well as bioethical. In many regards, positive results on the uniform panel’s secondary list of conditions (disorders that are not purposefully being tested for on the “core” universal recommended panel but can be detected through the differential diagnosis of a core condition) in the current NBS paradigm represent a construct to incidental findings that will become more prevalent should comprehensive genomics be used to determine NBS results that will be transmitted to families. The large amount of information generated, with much yet unknown as to what is clinically relevant or actionable, will confound the difficulty in reporting positive findings. Hence, the even-more-important need to encourage empirically informed research on attachment difficulties that is grounded in theory. 

The inevitable generation of incidental, collateral secondary information with next-generation sequencing compounds the ethical dilemmas expressed when expanded NBS by mass spectrometry began. By addressing the central challenges witnessed historically, with a perspective grounded in developmental theory, perhaps we can more objectively look ahead at the ethical implications of public health initiatives on the attachment process, given the likelihood that NBS will be further expanded into a high level of conditions reported with gene sequencing.

For parents receiving NBS findings that require further evaluation, NBS becomes a process rather than a specific test. How the information is conveyed—whether at a scheduled routine appointment, at an immediate additional appointment, or by mail, email, or phone—is integral to how families react psychologically to the information. To better assess the current short- and long-term impact of NBS disclosures on parental anxiety and interactions with infants influencing attachment, we need to delineate studies that base their conclusions on former means of communicating results. It is important to contrast and compare both the evolution and vicissitudes of various communicative methods that are actually used throughout the reporting processes in order to evaluate optimal strategies of communication to contribute to the establishment of better practices today—before NBS moves into fully comprehensive genomic sequencing in the public health domain. Many fundamental questions must be considered when heterogeneous incidental information, vastly beyond that generated by current NBS, is recognized to be not-so-incidental to families. The genomic analysis and provision for disclosing such large-scale, potentially significant secondary findings (including those equated with carrier status in parents) is untenable unless we foster improved strategies for communicating the information to families, which, by necessity, must encompass prior education and adequate genetic counseling and access to healthcare regardless of economic status. 

In addition, the robustness and uncertainty of expanded NBS panels can be offset by increasing efforts to explain the process of NBS and its reporting of results during pregnancy, as well as, should positive findings appear, the support of the genetic counselor or a knowledgeable healthcare provider. This call for services mirrors Faden and colleagues’ recommendation four decades ago. It is striking that, although comments have been made repeatedly suggesting that improved communication can potentially diminish parental anxiety, much remains to be accomplished. 

We conclude with questions, such as why are we still so far afield from providing these services and communication and why have researchers refrained from trying to identify which families, upon receiving positive screening findings, may be most at risk for reacting with a significant level of anxiety and/or depression of a long-standing nature that could disrupt the attachment process pathologically? Hopefully, studies in the future will be directed to improving communication-counseling with a more advanced understanding of the implications of positive NBS findings. As conditions propagate, this will further reduce the burdens on families as well as care providers and optimize opportunities for improved communication.

## Data Availability

No new data were created. This is an Opinion piece.
